# Frequency-specific brain network architecture in resting-state fMRI

**DOI:** 10.1038/s41598-023-29321-5

**Published:** 2023-02-20

**Authors:** Shogo Kajimura, Daniel Margulies, Jonathan Smallwood

**Affiliations:** 1 Faculty of Information and Brain Sciences, Kyoto Institute of Technology, Matsugasaki, Sakyo-ku, Kyoto, 606-8585 Japan; 2 Integrative Neuroscience and Cognition Center, Centre National de la Recherche Scientifique (CNRS) and Université de Paris, 75006 Paris, France; 3 Nuffield Department of Clinical Neurosciences, Wellcome Centre for Integrative Neuroimaging, University of Oxford, Oxford, OX3 9DU UK; 4 Department of Psychology, Queen’s University, Kingston, ON Canada

**Keywords:** Cognitive neuroscience, Social neuroscience

## Abstract

The analysis of brain function in resting-state network (RSN) models, ascertained through the functional connectivity pattern of resting-state functional magnetic resonance imaging (rs-fMRI), is sufficiently powerful for studying large-scale functional integration of the brain. However, in RSN-based research, the network architecture has been regarded as the same through different frequency bands. Thus, here, we aimed to examined whether the network architecture changes with frequency. The blood oxygen level-dependent (BOLD) signal was decomposed into four frequency bands—ranging from 0.007 to 0.438 Hz—and the clustering algorithm was applied to each of them. The best clustering number was selected for each frequency band based on the overlap ratio with task activation maps. The results demonstrated that resting-state BOLD signals exhibited frequency-specific network architecture; that is, the networks finely subdivided in the lower frequency bands were integrated into fewer networks in higher frequency bands rather than reconfigured, and the default mode network and networks related to perception had sufficiently strong architecture to survive in an environment with a lower signal-to-noise ratio. These findings provide a novel framework to enable improved understanding of brain function through the multiband frequency analysis of ultra-slow rs-fMRI data.

## Introduction

At rest, the brain can be organized into patterns of temporally organized activity, known as resting-state networks (RSNs), which can be visualized using functional magnetic resonance imaging (fMRI). These networks show a reasonable correspondence to the activation patterns observed when participants perform a variety of different tasks during fMRI acquisition^1–5^. Analysis of RSNs provides a powerful method to study large-scale functional integration of the brain^6–12^ as well as abnormalities associated with neurological and psychiatric diseases^13–18^.

Most research on resting-state functional networks has focused on an ultra-low frequency spectrum (typically around 0.01–0.1 Hz) of blood oxygen level-dependent (BOLD) signals because of their neurophysiological properties^19^ and the slow acquisition time resolution of fMRI. However, recent studies have shown that RSNs can also be observed in the relatively higher frequency spectrum present in the BOLD signal, that is, over 0.1 Hz up to 0.75 Hz, obtained using the multiband fMRI technique^20^. Baria et al.^21^ studied the spatial distribution of the BOLD signal as a function of four frequency bands and identified the frequency-dependent organizational patterns for both the whole brain and for specific RSNs. They found that the power spectral density distribution for the BOLD signal shifted from low to high frequencies along the visual ventral stream and suggested that this corresponds to the hypothesized increasingly abstract processes along this hierarchy. Additionally, the power spectral density shift pattern was heterogeneous across the regions within the default mode network (DMN)^19,22,23^, which is where all the streams converge, including the visual ventral stream^2^. Frequency specificity has also been reflected in the spatial distribution^24^, inter-network integration^25^, and task-related modulation^26^ of functional networks.

In studies of the frequency-specific function of RSNs, the network architecture is often assumed to be identical across frequency bands^21,24,26–30^, although there is a difference in whether the network architecture is pre-defined^24,28,30,31^ or defined in the study^21,26,27,29^. The assumption that the RSN architecture is similar among different frequency bands could lead to misinterpretation of the results and prevent an accurate understanding of the higher frequency band. Kajimura et al.^30,31^ reported the value of the higher frequency band; only the relatively higher frequency band data contained information regarding the initial feeling of compatibility between opposite-sex individuals. They decomposed the rs-fMRI BOLD signal into four frequency bands and applied a machine learning algorithm whose features comprised a newly defined connectome similarity, which is a vector of absolute Euclidean distances between the functional connectomes of two individuals, calculated from rs-fMRI data. As a result, the prediction compatibility was significantly higher than chance level only when the connectome similarity of the relatively highest frequency band was used as a feature. They also interpreted the result from the perspective of RSN-level contribution; however, the RSNs they applied were defined through lower frequency band data^5^, and multiple comparison corrections were applied. If the RSN architecture in the higher frequency band is significantly different from that in the lower frequency band, the findings should be reinterpreted in association with the appropriate definition of the RSN architecture.

Regarding the network architecture, some previous studies have indicated that changes in the network architecture according to frequency band while others have not. Baria et al.^21^ analyzed BOLD oscillations in full bandwidth and revealed that the spatial structure of power spectral density is significantly different across frequency bands both in the whole brain and in the DMN, indicating that the network architecture is reconfigured in higher frequency bands. Additionally, Wu et al.^32^ found a distance-frequency relationship that describes the decay of functional connectivity between distant regions in the higher frequency bands. This relationship might induce the reconfiguration of networks from distributed to local architectures in higher frequency bands. In contrast, Gohel and Biswal^33^ assessed the presence of RSNs across multiple frequency bands by fixing the number of networks and found that commonly known RSNs, such as the default mode, frontoparietal, dorsal attention, and visual networks, were consistently observed at multiple frequency bands. This result indicates that the RSNs found in the lower frequency bands also exist in the higher frequency bands. Independent of the competing nature of the network architecture in higher frequency bands (consistency or reconfiguration), the appropriate number of networks may vary across frequency bands. Incidentally, since the application of the maximal overlap discrete wavelet transform (MODWT) in research that examined the frequency-dependence of the brain’s functional properties for the first time^34^, it has become a major method for frequency decomposition in the field of neuroimaging research^35–40^; this is because of its suitability for the analysis of fractal signals or signals with 1/*f* processes, such as those associated with resting-state fMRI time-series^41^.

The current study examined whether the frequency at which RSNs are defined influences the architecture and resolution of the systems. As the signal-to-noise ratio decreases in higher frequency bands^24^, we assessed the impact of frequency by comparing the architecture of high-resolution RSNs (i.e., 17 RSNs based on Yeo et al.^5^) across frequency bands. If an increase in signal-to-noise ratio disturbs the detection of network architecture, the RSNs in higher frequency bands would be fragmented and would overlap less with the RSNs in lower frequency bands. Additionally, we re-analyzed the data to which the lower frequency-based RSN architecture were originally applied in order to interpret the results in relation to higher frequency data^30,31^. This enabled the data to be reinterpreted with an appropriate RSN architecture and potentially unveil the actual function of brain networks in higher frequency band data. For the method of frequency decomposition and the definition of functional networks, we utilized the MODWT and the clustering algorithm with a von Mises-Fisher distribution, respectively. We utilized the clustering algorithm with a von Mises-Fisher distribution, with which Yeo et al.^5^ provided one of the most influential definitions of the brain’s functional network organization. This method was also used to obtain the subject-specific network organization^42^.

## Methods

### NKI-Rockland sample data

We used data from the publicly available NKI-Rockland Sample (http://fcon_1000.projects.nitrc.org/indi/pro/nki.html) from the Nathan Kline Institute (NKI; Orangeburg, NY, USA) consisting of *N* = 140 (61 men; age = 39.2 $\pm$ 18.8 years), of whom, 120 were right-hand dominant (assessed with the Edinburgh Handedness Questionnaire^43^) and 61 reported health problems. Institutional Review Board Approval was obtained for this project at the Nathan Kline Institute (Phase I #226781 and Phase II #239708) and at Montclair State University (Phase I #000983A and Phase II #000983B). All research was performed in accordance with relevant guidelines/regulations. Written informed consent was obtained for all study participants. The fMRI data were acquired using a Siemens TrioTM 3.0 Tesla MRI scanner (Siemens, Erlangen, Germany) with an echo-planar imaging (EPI) sequence and the following parameters: repetition time (TR)/echo time (TE) = 645/30 ms, flip angle (FA) = 60$^\circ$, field of view (FOV) = 222 $\times$ 222 mm, voxel size = 3.0 $\times$ 3.0 $\times$ 3.0 mm, distance factor = 0%, number of slices = 40, multiband acceleration factor = 4. Each scan session was 10 min long and comprised 900 volumes. Physiological data (cardiac and respiratory recordings) were also obtained during scanning. T1-weighted images were acquired using the following magnetization-prepared rapid gradient echo (MPRAGE) sequence: TR/TE = 1900/2.52 ms, inversion time = 900 ms, FA = 9$^\circ$, FOV = 250 $\times$ 250 mm, voxel size = 1.0 $\times$ 1.0 $\times$ 1.0 mm, number of slices = 176.

### fMRI data preprocessing

All preprocessing was performed using the Data Processing Assistant for Resting-State fMRI Advanced Edition (DPARSFA; http://www.rfmri.org/DPARSF)^44^, which is based on the Statistical Parametric Mapping 8 software (SPM8; http://www.fil.ion.ucl.ac.uk/spm) and the resting-state fMRI Data Analysis Toolkit (REST; http://www.restfmri.net)^45^. First, the image time series for each participant was realigned using a six-parameter rigid-body transformation. We then performed slice-timing correction. All volume slices were corrected for differences in the signal acquisition time by shifting the signal measured in each slice relative to the acquisition of the slice at the first point of each TR. Individual structural (T1-weighted MPRAGE) images were co-registered to the mean functional image using a rigid-body transformation. The transformed structural images were then segmented into gray matter, white matter, and cerebrospinal fluid (CSF)^46^. Thereafter, we conducted nuisance covariate regression in the native space to minimize the effects of noise caused by the cardiac and respiratory cycles, scanner drifts, and motion. Nuisance regressors consisted of six motion parameters of the current volume and the preceding volume, plus each of these values squared, average white matter, CSF signals, global signal (GSR; as there is controversy about application of the GSR, we also conducted an identical analysis of the data without GSR). Furthermore, the cardiac and respiratory cycles measured by peripheral recordings were modeled with RETROICOR^47^ using the MATLAB PhysIO toolbox^48^ and included in the nuisance covariate regression model. In brief, this method assumes that the physiological processes are quasi-periodic^47^ and models such processes via a Fourier expansion of physiological phases^48^. This procedure enabled us to reduce the physiological noises in higher than traditional frequency ranges (cardiac fluctuation spectrum: around 0.2 Hz; respiratory spectrum: 0.1–0.15 Hz)^47,49^. Subsequently, the functional scans were spatially normalized to the Montreal Neurological Institute (MNI) stereotactic standard space and smoothed with a 6-mm full-width at half-maximum Gaussian kernel using the Diffeomorphic Anatomical Registration Through Exponentiated Lie Algebra (DARTEL) toolbox^50^.

### Parcellation of the cerebral cortex

To reduce the computational cost of the frequency decomposition and clustering analysis approaches, we divided the Yeo’s gray matter template^5^ into sub-regions using the Ncut random parcellation algorithm with the python package pyClusterROI^51^. We set the maximum number of regions as 3000 for the algorithm and obtained 2143 cortical sub-regions. Using this definition of sub-regions, the subsequent procedures extracted the BOLD signal from these regions instead of each voxel.

### Wavelet decomposition and multiband frequency definition

We utilized the MODWT to define the multiband frequency^34^. For each participant, all BOLD signals extracted from the 2143 sub-regions were decomposed by MODWT into seven levels. The Daubechies Least Asymmetric 14 was chosen for the wavelet filter and length, based on practical guidelines^52^. Then, the fast Fourier transform was applied to the decomposed signals and the minimum and maximum frequency across regions were obtained for each participant. To define participant-independent multiple frequency bands, we obtained the averages of minimum and maximum frequency for each level of decomposition. Finally, four frequency bands were defined so that the current result was comparable and applicable to previous research^21,24,26–30^: 0.007–0.052 Hz (the three lower levels were out of the scope and thus integrated), 0.052–0.106 Hz, 0.106–0.206 Hz, and 0.206–0.438 Hz (the seventh level was out of measurement range and thus discarded; the same frequency bands were defined for the data analyzed without GSR). These frequency bands were denoted as F1, F2, F3, and F4, as required. Finally, the BOLD signals without MODWT decomposition were bandpass filtered for each frequency band.

### Clustering

We applied the clustering approach of Yeo et al.^5^ to define the boundaries of functionally distinct cortical networks as sets of cortical regions with similar profiles of corticocortical functional connectivity in each frequency band. For each participant, we computed the Pearson’s correlation coefficient between the fMRI time series of the 2143 sub-regions and the time series of 8570 voxels that, for computational efficiency, comprised approximately 4 voxels from every sub-region instead of each individual voxel. Binarization of the correlation matrix leads to significantly better clustering results, although the algorithm appears robust to the particular choice of the threshold. Thus, we binarized the 2,143 $\times$ 8,570 matrix of correlation coefficients for each participant by keeping the top $x$% ($x=\mathrm{1,2},\dots ,10$) of the correlation coefficients, and averaged the matrices across participants for each threshold for the subsequent clustering algorithm.

Following Yeo et al.^5^, the clustering algorithm employed in this study modeled the data with a von Mises-Fisher distribution^53^. The data were modeled as 8570 points on a 2143-dimensional unit hypersphere embedded in a 2143-dimensional Euclidean space, where the distances between points were measured by their geodesic distance on the hypersphere. Clustering aims to group sub-regions that are close together on a non-Euclidean canvas (i.e., those that have similar connectivity profiles) into the same cluster. Measuring distances between points using their geodesic distances is equivalent to defining the similarity between two correlation profiles as the correlation between the correlation profiles.

### Determination of the appropriate number of clusters for each frequency band

As the clustering algorithm requires the user to select the required number of clusters a priori, we applied the algorithm with 3–20 numbers of clusters and assessed the results from the perspective of overlap with task-evoked activations provided by Neurosynth^54^. As reported by previous studies^55–57^, this approach enables the detection of functionally reasonable network architectures. The 24-task activation masks (e.g., attention, cognitive control, learning, reward, theory of mind, and working memory; detailed in the Supplementary Information) covered 68.4% of the gray matter mask used in the current study (Fig. S1). In each frequency band, the overlap score for a cluster number for each threshold was calculated as shown in Eq. (1):

1 $${RC}_{tk}=\frac{1}{N}\sum_{i=1}^{N}\underset{j}{\mathrm{max}}\left(\frac{{Ma}_{i}\cap {Mc}_{tj}}{{Ma}_{i}}\times \frac{{Ma}_{i}\cap {Mc}_{tj}}{{Mc}_{tj}}\right) (j=1,\dots ,k)$$

where *Ma* and *Mc* are the task activation mask and the mask of a cluster, respectively, and *t*, *k*, and *N* are the threshold value, number of clusters, and number of task activation masks (i.e., 24), respectively. The cluster number that had the largest *RC*_*tk*_ most frequently across the thresholds was defined as the best clustering result for each frequency. The network architecture was defined by *k*-means cluster analysis of the average adjacency matrices for the thresholds that had the largest *RC*_*tk*_ for the best cluster number.

### Re-analysis of the data originally interpreted based on lower frequency-based RSN architecture

Previously, Kajimura et al.^30,31^ decomposed the BOLD signals from rs-fMRI into four frequency bands (0.014–0.027 Hz, 0.027–0.055 Hz, 0.055–0.109 Hz, and 0.109–0.199 Hz) and applied a machine learning algorithm to predict the compatibility of opposite-sex individuals. The predictive accuracy was significantly higher than chance only when the highest frequency-based feature was used. They also analyzed the data from the perspective of RSN-level contribution using a lower frequency-based RSN architecture^5^, which comprised seven networks (the visual network, somatosensory-motor network, salience network, executive control network, DMN, limbic system, and cerebellum), and found a significant overall positive and negative contribution of the cerebellum.

We re-analyzed the data using the newly defined RSN architecture of F3 (0.106–0.206 Hz), which is the frequency band most similar to the one with significant predictive ability identified in the original data (i.e., 0.109–0.199 Hz^30,31^). To test the statistical significance of the network level, laterality within network-level contributions, and inter-network-level contributions, we performed exploratory analyses using permutation tests. In the permutation tests, the ROI labels were randomized, and each index was calculated 1000 times to assess whether the result was significant under the null distribution. The network-level contribution was defined as the total number of features that contributed to the prediction in which the ROIs in a given network were involved. The laterality within the network-level contribution was defined as the difference in the number of features contributing to the prediction between the right and left hemispheres in a given network. The inter-network-level contribution was defined as the total number of features contributing to the prediction in which ROIs in a given pair of networks were involved.

## Results

### Appropriate number of clusters for each frequency band

**Figure 1:**
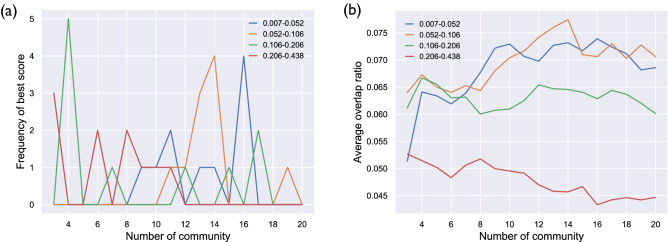
(**a**) The frequency of the best overlap score for each cluster number and each frequency band. (**b**) Averages of overlap scores across thresholds for each cluster number and each frequency band.

Figure 1a–b shows the frequency of being the best overlap score for each cluster number and each frequency band, and the averages of overlap scores across thresholds, respectively (the data without GSR are shown in Fig. S2a–b). The cluster numbers that most frequently exhibited the best overlap score were 16 for F1, 13 for F2, 4 for F3, and 3 for F4 (for the data without GSR, the cluster number that most frequently had the best overlap score was 17 for F1, 15 for F2, 4 for F3, and 3 for F4).

### RSN architecture for each frequency band

**Figure 2:**
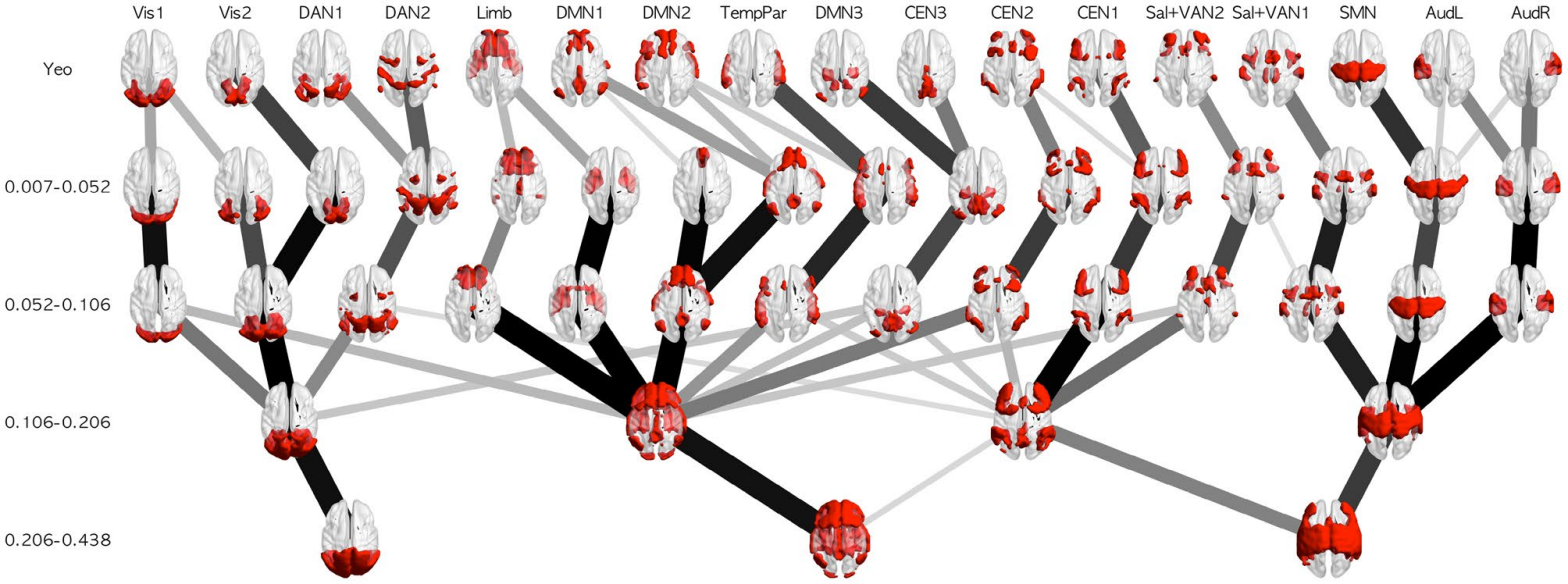
Network architectures from the data of Yeo et al.^5^ and each frequency band. The line width and color reflect the overlap ratio between networks. This figure was created using Matlab 2017a (Mathworks, USA) with BrainNet Viewer 1.7^58^ and python 3.6.0 with scikit-image 0.19.3, Pillow 9.4.0, and matplotlib 3.6.3.

The network architectures are illustrated in Fig. 2 (the network structures obtained from the data without GSR are displayed in Fig. S3). The most subdivided networks (i.e., networks of F1) were labeled based on similarity with the network of Yeo et al.^5^ and visual inspection. The line width reflects the overlap ratio between the networks. Most of the networks in F1 had an architecture similar to that of Yeo’s networks, validating the current methodology. Some exceptions were found: the DAN, DMN, and auditory networks divided into subnetworks in Yeo’s networks were merged into a single network or two subnetworks, and the limbic network was divided into the orbitofrontal cortices (OFC) and anterior temporal lobules (ATL). Additionally, one of the CEN and DMN subnetworks was merged into a single network. These differences could be explained by the difference in the frequency range with or without physiological noise regression.

In F3, the subdivided networks in F1 and F2 were mainly integrated into VN_F3_, SMN_F3_, CEN_F3_, and DMN_F3_ rather than reconfigured; the DAN was integrated into VN_F3_, Sal + VAN subnetworks were integrated into SMN_F3_ and CEN_F3_, respectively, and CEN3 + DMN3 was split into VN_F3_, CEN_F3_, and DMN_F3_. CEN2 was mainly absorbed by DMN_F3_ rather than CEN_F3_. DMN_F3_ absorbed not only the DMN subnetworks and CEN but also a part of the VN, Sal + VAN, OFC, and ATL. In F4, VN_F4_ and DMN_F4_ virtually had the same architecture as the corresponding network in F3, whereas SMN_F3_ and CEN_F3_ were integrated into a single network (SMN + CEN_F4_). These results indicate that in the higher frequency bands, the roughly divided networks integrate the functions that were processed by finely specialized subnetworks in the lower frequency bands. In addition, DMN_F4_ was the only complex network that survived without integration with primary networks. This could reflect the geometric and biophysiological distance between the DMN and primary networks^2^ as well as the consistent specialized function of the DMN across different frequency bands.

### Comparison of the architecture of high-resolution RSNs across frequency bands

**Figure 3:**
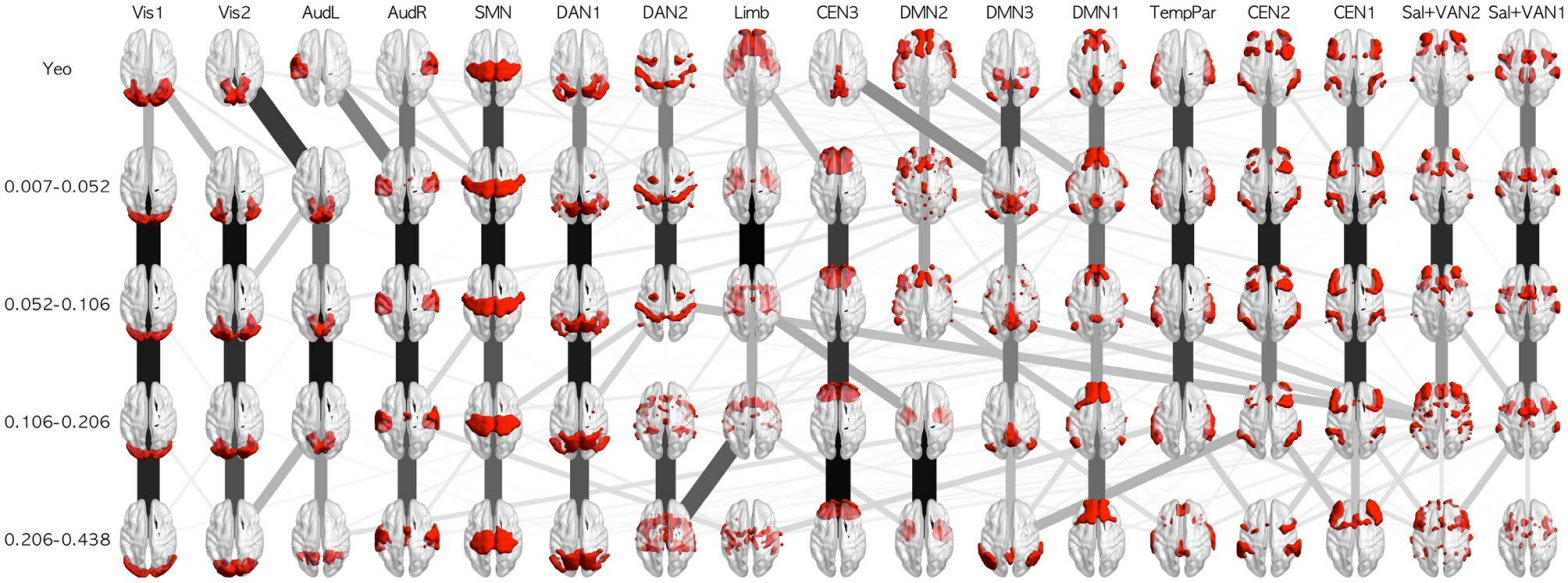
Seventeen RSNs from the data of Yeo et al.^5^ and each frequency band. The line width and color reflect the overlap ratio between networks. This figure was created using Matlab 2017a (Mathworks, USA) with BrainNet Viewer 1.7^58^ and python 3.6.0 with scikit-image 0.19.3, Pillow 9.4.0, and matplotlib 3.6.3.

To assess whether the remarkably smaller number of RSNs in F3 and F4 reflect the actual functional architecture or just the lower signal-to-noise ratio of the BOLD signal in higher frequency bands^24^, we compared the architecture of high-resolution RSNs (i.e., 17 RSNs based on Yeo et al.^5^) across frequency bands. As shown in Fig. 3, most of the networks detected in F1 and F2 were also detected in F3, which indicates that at least the smaller number of RSNs in F3 was not due to the lower signal-to-noise ratio but rather reflected the functional architecture. Regarding F4, central executive and attention networks were fragmented and were not consistent with the RSNs in the other frequency bands, indicating that the lower signal-to-noise ratio disturbed the detection of the central executive and attention networks in this frequency band. In addition, the DMN and networks related to perception were detected even in F4, indicating that these networks have sufficiently strong architectures and substantial functions to survive such an environment. Furthermore, combined with the result of F3, the smallest number of RSNs might actually reflect the functional architecture rather than just a failure of detection.

### Re-analysis of the data originally interpreted based on lower frequency-based RSN architecture

Regarding the network-level contribution, the cerebellum showed significant overall positive (*p* = 0.032 after FDR correction) and negative (*p* = 0.005 after FDR correction) contributions, indicating the same results as in the original study. The re-analysis revealed further significant results, which were not apparent in the original analysis using the lower frequency-based RSN architecture^30,31^: the significant negative contribution of the cerebellum was right hemisphere-dominant (*p* = 0.050 after FDR correction), and the inter-network contribution of the cerebellum and limbic areas was significantly positive (*p* = 0.018 after FDR correction).

## Discussion

The brain functional network architecture in the resting-state ultra-low BOLD signal has undoubtedly contributed to the understanding of brain function and of abnormalities associated with neurological and psychiatric diseases. Its importance is further emphasized by the fact that network architecture-based analysis is frequently used in contemporary research on the frequency-specific properties of BOLD signals. However, previous studies have been conducted on the premise that network architecture is identical across frequency bands, and the possibility that the network architecture itself is frequency-specific has never been questioned. The current research is the first to demonstrate that:

1. The resting-state BOLD signals that split into multiple frequency bands have frequency-specific network architecture; that is, the subnetworks finely subdivided for the lower frequency bands are integrated into fewer networks for relatively higher frequency bands rather than reconfigured.
2. The DMN and networks related to perception have sufficiently strong architecture to survive the increasing signal-to-noise ratio following an increase in frequency.
3. The smaller number of RSNs in the higher frequency bands may reflect the functional architecture rather than the lower signal-to-noise ratio.
4. The appropriate RSN architecture for higher frequency bands revealed the higher frequency-specific function that was not observed when applying the lower frequency-based RSN architecture.

Although there has been increasing interest in the multiband information of resting-state BOLD signals^21,24,26–29^, the quality of information included in the higher frequency bands remains largely unknown. The current study is the first to reveal that there are also network architectures in the higher frequency bands, which are comparable with those in the lower frequency bands; a relatively high-frequency resting-state BOLD signal, which has been neglected in most rs-fMRI research, has sufficient function-related information to detect meaningful networks and is worth further investigation to better understand brain function and abnormalities associated with neurological and psychiatric diseases from the perspective of brain network architecture.

The elucidation of network architecture by the current study has the potential to help improve future research on multiple frequency band analysis. The results provide a standard for the selection of networks of interest and inter-network interactions for the corresponding frequency bands. In addition, as the networks in higher frequency bands (i.e., the visual network, SMN, CEN, and DMN) have comparable or corresponding architectures with subdivided networks in lower frequency bands, it would be beneficial to define an identical network architecture through all frequency bands to compare the functional properties across frequency bands. This may improve the clarity of interpretation of the results. As the optimal number of networks was determined through fit to the activation maps of various tasks, the significant decrease in network resolution in the higher frequency bands indicates that there is insufficient task-related information to enable improvement of the fit to task activation. In such instances, applying the network architecture with appropriate resolution to higher frequency band data may reduce the likelihood of false positive results or the misinterpretation of meaningful results that may occur when a finely subdivided network architecture is applied.

Only the DMN remained independent from the primary networks (i.e., the visual network and SMN), even in the highest frequency band. Although it is unclear to what extent the convergence of network architecture in higher frequency bands is affected by the decrement of the signal-to-noise ratio, the observed results are capable of reflecting neurophysiological constraints. The boundary between the DMN and primary networks corresponds to a geometric property^2^; core regions of the DMN (e.g., the medial prefrontal cortex, posterior cingulate cortex, and angular gyrus) are equidistant from the primary regions and at the other end in the principal gradient of functional connectivity in the low-frequency band^2^. Considering the consistent presence of the DMN across frequency bands, the resting-state BOLD signal might embed information processing of the DMN for various complex socio-cognitive functions across a broad range of frequencies. Future studies are required to better understand the exact DMN function from the perspective of multiband frequencies.

The pattern of network convergence, that is, finely subdivided networks in lower frequency bands, was integrated with geometrically adjacent networks in higher frequency bands, supporting the distance-frequency relationship that describes the decay of functional connectivity between distant regions in higher frequency bands^32^. Regarding the two networks associated with Sal + VAN in F2, one was composed of regions around the central sulcus and was integrated with the adjacent SMN, while the other was composed of prefrontal regions and was integrated with the adjacent CEN in F3. The DAN in F2 was also integrated with the adjacent visual network. Referring to the hypothesis advocated by Wu et al.^32^, the convergence might be attributed to a larger attenuation of synchrony of brain regions separated by longer distances and/or connected with more synaptic steps. It would be beneficial to investigate the relationship between this geometric/physiological constraint and the frequency-specific function of networks in future studies.

The current study also demonstrated that re-analyzing previous data on higher frequency functions with appropriate RSN architecture unveils the higher frequency-specific functions that were not observed when applying the lower frequency-based RSN architecture. In previous studies on the frequency-specific function of RSNs, the network architecture was regarded as identical across frequency bands^21,24,26–30^. Both previous and future research could benefit from the frequency-specific RSN architectures found in the current study, as they could reveal higher frequency-specific network functions that might be involved in important socio-cognitive functions^30,31^. However, to maximize the benefits, it is necessary to establish a frequency-specific RSN architecture through the conduct of replication studies in other populations.

Although the network architecture in the higher frequency bands (F3 and F4) was almost the same between the data with and without GSR, a difference in the lower frequency bands (F1 and F2) was noticeable. Visual inspection indicated that the network architecture of F1 was more stable in the data with GSR (Fig. 2) than in those without GSR (Fig. S3). In the architecture of F1 detected by the data excluding GSR, the networks that correspond to the DMN_2_ and CEN_2_ of Yeo et al.^5^ are partially asymmetric and, thus, appear unstable. This indicates that GSR enabled us to obtain better network architecture. The purpose of GSR is to remove the global signals that contain many confounds arising from motion, cardiac and respiratory cycles, arterial CO_2_ concentration, blood pressure/cerebral autoregulation, and vasomotion^59,60^. Of note, GSR enhances the detection of system-specific (anti-)correlations^61^, which might differentiate the network detection results. Although a previous study, which used the same clustering method as did the current study, did not apply GSR^5^, their sample size was far larger than that of the current study (1000 vs. 140). Thus, the use of GSR may compensate for small sample sizes.

Although the MODWT is often used for the frequency decomposition of fMRI time-series due to the compatibility between the two^34–40^, the selection of frequency bands is determined methodologically and not on a physiological basis. This is a limitation of our study. Indeed, the RSN architecture was similar between F1 and F2, indicating that they carry largely overlapping information. Further research on the higher frequency components of the BOLD signal could reveal more physiologically plausible frequency bands in order to maximize the obtainable physiological information.

In conclusion, this study investigated the frequency-specific network architectures of multiband rs-fMRI data, revealing that finely divided networks in lower frequency bands were integrated into a few networks that maintained a functional structure. In particular, the DMN was independent of primary networks in the highest frequency bands, indicating that DMN information processing was associated with a wide range of functions distributed across frequency bands. These findings emphasize the importance of the appropriate incorporation of information from the higher frequency bands of ultra-slow rs-fMRI data, which has been discarded in most rs-fMRI studies. Our study also provides a novel framework for multiband frequency analysis that facilitates a better understanding of brain function.

## Supplementary Information

Supplementary Information.

## Data Availability

The datasets (NKI-Rockland Sample) used during the current study provided by the Nathan Kline Institute (NKI, NY, USA) are available in the homepage (http://fcon_1000.projects.nitrc.org/indi/pro/nki.html).
