# Supplementary material for: Frequency-specific brain network architecture in resting-state fMRI
Source: Sci Rep. 2023 Feb 20;13:2964. doi: 10.1038/s41598-023-29321-5 (PMC9941507; doi:10.1038/s41598-023-29321-5)
Supplement: Supplementary file 1 — Supplementary Information. [file 41598_2023_29321_MOESM1_ESM.docx]

Supplementary

The 24 task activation masks for determining appropriate number of the clustering were collected from the result of “Term-based meta-analyses” provided by neurosynth (<https://neurosynth.org/analyses/terms/>). The terms were attention, auditory, choise, conflict, decision, emotional, episodic memory, executive, impulsivity, inhibition, integration, language, learning, motor, pain, perception, reward, self referential, semantic, sensory, theory of mind, valence, visual, and working memory.

Figure S1. (a) The areas overlapping with any of task activation masks provided by neurosynth^1^. The areas where do not have any overlap are colored black. (b) The coverage of the networks of Yeo et al. (2011)^2^. This figure was created using Matlab 2017a (Mathworks, USA) and BrainNet Viewer 1.7^3^.

Figure S2. (a) The frequency of being the best overlap score for each cluster number and each frequency-band. (b) Averages of overlap scores across thresholds for each cluster number and each frequency-band.





Figure S3. Network architectures from the data of Yeo et al. (2011)^2^ and each frequency band of the data without GSR. The line width and color reflect the overlap ratio between networks. This figure was created using Matlab 2017a (Mathworks, USA) with BrainNet Viewer 1.7^3^ and python 3.6.0 with scikit-image 0.19.3, Pillow 9.4.0, and matplotlib 3.6.3.

**Reference**

1. Yarkoni T, Poldrack RA, Nichols TE, Van Essen DC, Wager TD. Large-scale automated synthesis of human functional neuroimaging data. *Nat Methods*. 2011;8(8):665-670. doi:10.1038/nmeth.1635

2. Yeo T, Krienen FM, Sepulcre J, et al. The organization of the human cerebral cortex estimated by intrinsic functional connectivity. *J Neurophysiol*. 2011;106(3):1125-1165. doi:10.1152/jn.00338.2011

3. Xia M, Wang J, He Y. BrainNet Viewer: A Network Visualization Tool for Human Brain Connectomics. *PLoS One*. 2013;8(7). doi:10.1371/journal.pone.0068910
